# An Approach for Engineering Peptides for Competitive Inhibition of the SARS-COV-2 Spike Protein

**DOI:** 10.3390/molecules29071577

**Published:** 2024-04-01

**Authors:** Ana Paula de Abreu, Frederico Chaves Carvalho, Diego Mariano, Luana Luiza Bastos, Juliana Rodrigues Pereira Silva, Leandro Morais de Oliveira, Raquel C. de Melo-Minardi, Adriano de Paula Sabino

**Affiliations:** 1 Laboratory of Bioinformatics and Systems, Department of Computer Science, Institute of Exact Sciences, Universidade Federal de Minas Gerais, Belo Horizonte 31270-901, Brazil; apabreu@ufmg.br (A.P.d.A.); fredericochaves@dcc.ufmg.br (F.C.C.); luizabastosluana@ufmg.br (L.L.B.); leandrobioinfo@ufmg.br (L.M.d.O.);; 2 Department of Biochemistry and Immunology, Institute of Biological Sciences, Universidade Federal de Minas Gerais, Belo Horizonte 31270-901, Brazil; 3 Laboratory of Clinical and Experimental Hematology, Clinical and Toxicological Analysis Department, Faculty of Pharmacy, Universidade Federal de Minas Gerais, Belo Horizonte 31270-901, Brazil

**Keywords:** COVID-19, SARS-CoV-2, peptides, docking, protein–peptide interactions, POTTER, molecular dynamics simulations

## Abstract

SARS-CoV-2 is the virus responsible for a respiratory disease called COVID-19 that devastated global public health. Since 2020, there has been an intense effort by the scientific community to develop safe and effective prophylactic and therapeutic agents against this disease. In this context, peptides have emerged as an alternative for inhibiting the causative agent. However, designing peptides that bind efficiently is still an open challenge. Here, we show an algorithm for peptide engineering. Our strategy consists of starting with a peptide whose structure is similar to the interaction region of the human ACE2 protein with the SPIKE protein, which is important for SARS-COV-2 infection. Our methodology is based on a genetic algorithm performing systematic steps of random mutation, protein–peptide docking (using the PyRosetta library) and selecting the best-optimized peptides based on the contacts made at the peptide–protein interface. We performed three case studies to evaluate the tool parameters and compared our results with proposals presented in the literature. Additionally, we performed molecular dynamics (MD) simulations (three systems, 200 ns each) to probe whether our suggested peptides could interact with the spike protein. Our results suggest that our methodology could be a good strategy for designing peptides.

## 1. Introduction

In early 2020, an outbreak of the SARS-CoV-2 (Severe Acute Respiratory Syndrome Coronavirus 2) virus, which was responsible for a respiratory disease called COVID-19, devastated global public health. So far, more than 6.9 million deaths and 771 million cases have been recorded worldwide (OMS) [1]. Its rapid international spread required the development of safe and effective prophylactic and therapeutic agents against the infection of its causative agent. The first step was to understand the mechanism of viral infection to develop intervention strategies against the new coronavirus [2].

SARS-CoV-2 is a coronavirus belonging to the *Coronaviridae* family. Its genome comprises a single-stranded RNA (+ssRNA) molecule containing approximately 30 kb [3,4]. The main genes in its genome include those that encode structural proteins, such as the spike (S) protein, as well as envelope (E), membrane (M), and nucleocapsid (N) proteins [5,6]. Highlighted is the S protein, a transmembrane glycoprotein that contains the S1 subunit and the S2 subunit and which binds to a transmembrane protease Serine Protease 2 (TMPRSS2), which is present in the host membrane [7,8]. As with other class I membrane fusion proteins, the spike protein is cleaved post-translationally—in this case, by the action of furin—into the S1 and S2 components, which remain associated after cleavage. The S1 subunit, which contains the receptor-binding domain (RBD), capable of functionally folding independently, can bind to angiotensin II-converting enzyme (ACE2) [8,9]. The S2 subunit, composed of the fusion peptide (FP), heptad 1 (HR1) and heptad 2 (HR2) repeat domains, the transmembrane domain (TM), and cytoplasmic domain (CP) fusion, is responsible for mediating fusion and viral entry [8,10].

The SARS-CoV-2 spike glycoprotein binds to the ACE2 homodimer, which facilitates the entry of the virus into host cells, forming the SARS-CoV-2-RBD/ACE2 interaction [11,12]. ACE2 is a single-pass type I transmembrane metallocarboxypeptidase enzyme responsible for the maturation of the peptide hormone angiotensin (Ang), which regulates vasoconstriction and blood pressure. It is present in type II alveolar epithelial cells and is also expressed in several extrapulmonary tissues, including the heart, kidney, and intestine [13]. Its structure is formed by two main domains—the N-terminal PD and the collectrin-like domain (CLD) at the C-terminal end. After binding to the ACE2 enzyme receptor, the virus particle uses host cell receptors and endosomes to enter cells. The host protease, TMPRSS2, facilitates entry into the cell through the S protein. Once inside the cell, viral polyproteins encoding the replicase-transcriptase complex are synthesized. The virus, in turn, synthesizes RNA through its RNA-dependent RNA polymerase. Structural proteins are synthesized, leading to the completion of the assembly and release of viral particles [12,14]. Therefore, the spike glycoprotein (S) performs the functions of binding, fusion, and viral entry and can be used as a target for the development of antibodies, entry inhibitors, and vaccines.

In this context, peptides have emerged as an alternative for inhibiting the causative agent. Peptides are molecules composed of short amino acid residues of 2–50 sequences linked by peptide bonds [15,16]. They play crucial roles in living beings, including cell signaling and immune modulation [17,18]. In therapeutics, peptides have several promising characteristics compared to small molecules and therapeutic proteins, including high structural compatibility with target proteins and the ability to disrupt protein–protein interfaces. They are also highly selective and effective while remaining relatively safe and well-tolerated at the same time. For these reasons, they have been used in studies of peptide-based vaccines, with several applications such as cancer and HIV and fewer applications for infectious viral diseases [18,19]. Therefore, they could be an alternative to propose vaccines or medicines to mitigate global pandemics, such as the recent COVID-19 pandemic. However, designing peptides that bind efficiently is still an open challenge.

In this study, we present an approach to designing peptides that could be used for target-based vaccine development called POTTER (peptide optimization tool to enhance receptor binding). We aimed to detect peptides that can interact with the spike (S) glycoprotein, preventing it from binding to the human angiotensin-converting enzyme 2 (ACE2). It is known that peptide-mediated interactions are gaining attention due to their predominant roles in the many regulatory processes involving dynamic interactions between proteins. The structures of such interactions provide an excellent starting point for their characterization and manipulation and may provide clues for targeted inhibitor design.

## 2. Results and Discussion

### 2.1. Algorithm Overview

In this section, we provide an overview of the POTTER algorithm. The algorithm aims to return an optimized peptide that can best bind to the SARS-COV-2 spike protein. Thus, as input (i), the algorithm received the initial peptide (we used the helix-alpha extracted from ACE2), and as input (ii), it received the initial receptor (the spike protein). Additionally, it receives the following three parameters as input:

- Generations (*G*): how often the process is repeated;
- Mutants (*M*): the number of mutants randomly generated in each generation;
- Repeats (*R*): the number of repeated dockings for each mutant.

Therefore, the following steps were executed: the peptide’s initial sequence was modeled using PyRosetta version 4—2022 [20]. Then, we performed docking of the peptide against the receptor *R* times. The pose of the peptide with the lowest PyRosetta score was selected.

**Figure 1:**
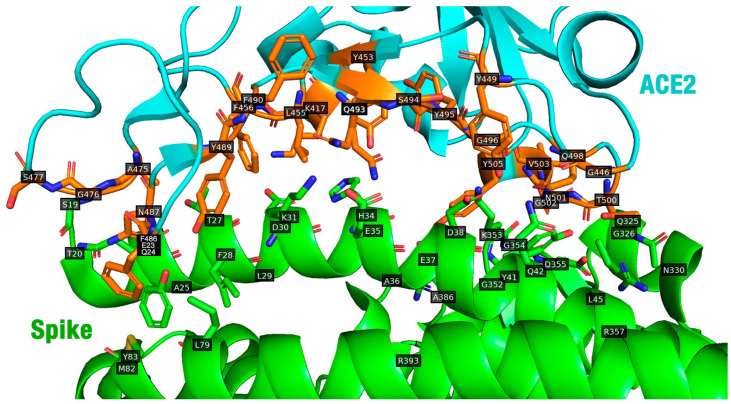
Interface interactions between ACE2 (cyan cartoon) and the spike receptor (green cartoon). Residues from ACE2 closer to the spike are shown as orange sticks (PDB ID: 6M0J). Blue: nitrogen atoms; red: oxygen atoms. The figure was generated using Open-Source PyMOL Version 2.5 (Schrödinger, LLC, New York, NY, USA).

Next, the “occupancy” of the selected pose was calculated. Occupancy is the percentage of peptide residues interacting with the receptor. This metric evaluates whether each of the peptide’s amino acids has at least one atom within 6 Å of one of the following receptor residues: K417, G446, Y449, Y453, L455, F456, A475, G476, S477, F486, N487, Y489, F490, Q493, S494, Y495, G496, Q498, T500, N501, G502, V503, or Y505 (Figure 1).

The algorithm was executed *G* times, with each execution being called a “generation”. Additionally, in the first generation, peptide residues that were more than 6 Å from the receptor were removed.

For each remaining generation (from 2 to G), *x* random mutations were made in the peptide structure. The value of *x* was defined randomly and could vary from one residue to the maximum size of the peptide sequence in generation two. This process was repeated *M* times, generating *M* mutants of the same size with random mutations. These mutants were included in a list called the “population”.

Table 1 demonstrates the allowable substitutions used throughout this study. The suggested options were based on conservative substitutions in evolution, which were accepted in secondary structure propensities [21,22]. They have been considered as alternative mutations in peptide design, i.e., amino acid residues with similar properties [21,22].

For each population element, the mutant peptide was modeled and docked against the receptor *R* times—in both cases, using PyRosetta. Note that all *R* poses were included in the next step (in steps 2 to G, the docking score was not used).

Next, the occupancy rate of the new complex and the contacts made between peptide and receptor were calculated. The following contacts were evaluated: (i) hydrophobic, (ii) aromatic stacking, (iii) attractive, (iv) hydrogen bond, (v) repulsive, and (vi) salt bridge contacts. Contacts were not used in the algorithm but were stored in a report that can be used for decision-making.

The peptide with the highest occupancy was selected as the starting point for the next generation. Then, the “population” list was deleted, and the process was repeated *G* times.

#### Algorithm Demonstration

**Figure 2:**
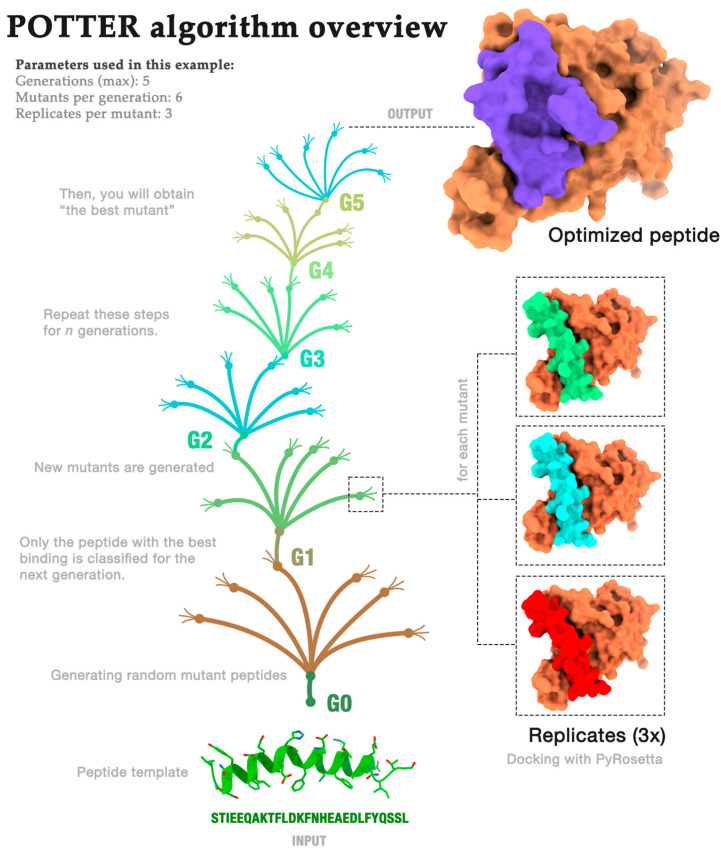
POTTER algorithm overview using the following parameters: G = 5, M = 6, and R = 3.

For instance, if we run POTTER using the parameters of generations (5), mutants per generation (6), and replicates per mutant (3), the algorithm would be executed similarly to the overview exhibited in Figure 2. First, the initial peptide is randomly mutated, generating six new structures. Each structure is then docked against the receptor (the spike protein) three times. Then, the algorithm calculates the contacts performed between each peptide residue (occupancy). The peptide with the highest occupancy percentage is selected for use in the next generation. This process is repeated for five generations. In the last generation, we hypothesize that we would obtain an optimized peptide that could bind better to the receptor and be a potential inhibitor.

To better understand how the algorithm works and its possible uses in peptide design, we conducted three case studies with different parameters, as discussed in the next section.

### 2.2. Case Studies

Initially, we analyzed the structure of the SARS-CoV-2 spike complexed with ACE2. Our initial objective was to design a peptide that would bind to the spike protein, competing with the binding that occurs with ACE2. We hypothesized that we could propose a peptide that binds to the spike protein by analyzing an experimentally obtained structure. Thus, the part of ACE2’s structure that binds to the spike protein would be a starting point. We used the PyMOL tool to detect the residues connecting both proteins. We noticed that the main residues interacting with the spike protein are in an alpha-helix. We thus extracted the sequence STIEEQAKTFLDKFNHEAEDLFYQSSL corresponding to this region. After extracting this sequence, we performed peptide modeling using ColabFold v1.5.5. The obtained structure will henceforth be called “peptide template”.

The Spike protein structure (E chain) was extracted from PDB 6M0J. Then, the peptide template was docked against the ACE2 structure using PyRosetta. Finally, the spike structure and the peptide template were used as input for the POTTER algorithm, which was run in three experiments with the following parameters: (i) replicates: 50; mutants: 50; generations: 17; (ii) replicates: 3; mutants: 100; generations: 100; (iii) replicates: 3; mutants: 1000; generations: 14.

For each experiment, we evaluated all generations and selected the mutants that presented the best binding with the spike protein. The program defines the best peptide using the percentage of residues making contact (higher occupancy—HO). However, many other factors can be used to choose the best peptide. The algorithm returns a series of metrics, such as contacts and docking score. Therefore, we performed a subsequent manual curation step to select the best peptide.

At this stage, experts filtered out complexes whose docking score returned by the PyRosetta tool was negative. They then manually analyzed each of the structures using the PyMOL tool. The peptide in a pose closest to the binding site was selected as the best pose by manual curation (MC). Additionally, we considered a case in which the best peptide was selected based on the Lowest Docking Score (LDS).

We can observe that, in some cases, the LDS peptide has a sequence similar to the MC peptide. In this case, the experts considered that the peptide was more appropriate to bind to the spike protein; however, they disagreed with the best pose indicated by the docking software.

Note that the sequences suggested by the POTTER algorithm have sequence similarities (identities) ranging between 17 and 57%. This denotes how important the choice of the initial peptide is in terms of suggesting the best peptide optimized according to the POTTER algorithm. Furthermore, it is notable how different input parameters produce peptides with quite different sequences. Therefore, when using the presented algorithm, it is necessary to test several parameters.

The results are summarized in Table 2.

### 2.3. Molecular Dynamics Simulations

To evaluate the proposed optimized peptides, we performed molecular dynamics (MD) simulations (details are included in Section 3). Due to restrictions imposed by the high computational costs of performing MD simulations, we chose only the complexes suggested in case study 1. We constructed three systems, namely (i) a spike protein complexed with the peptide template, (ii) a spike protein complexed with the lowest docking score (LDS) peptide, and (iii) a spike protein complexed with the higher occupancy (HO) peptide. We did not prepare a system for the MC peptide because it has the same sequence as the LDS peptide.

**Figure 3:**
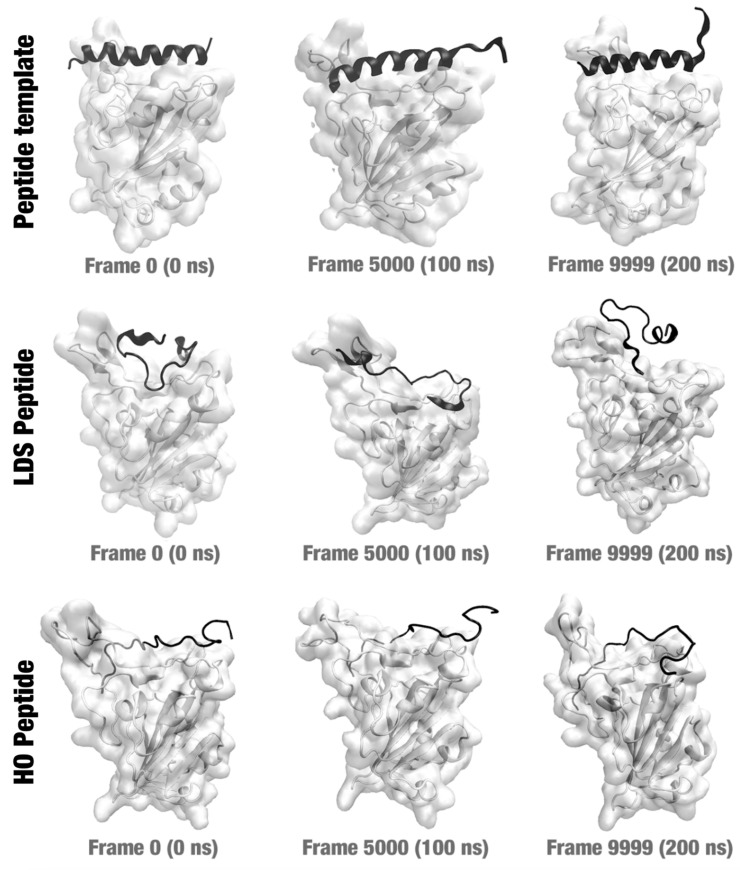
Frames 0 (0 ns), 5000 (100 ns), and 9999 (200 ns) from the three MD simulations: spike complexed with the peptide template (**top**); spike complexed with the LDS peptide (**middle**); and spike complexed with the HO peptide (**bottom**).

The MD simulation was initiated based on the hypothesis that the peptide was already connected to the spike protein binding region. Our objective was to verify whether the peptide would remain connected to the protein after a certain time (we arbitrarily set the time to 200 ns). Hence, we performed 200 ns (10,000 frames) of MD simulations for each system. Figure 3 illustrates three frames of each MD simulation (frame 0—the first; frame 5000—the middle; and frame 9999—the last). The peptide remained connected to the protein structure throughout the MD simulation period in all three cases.

We can see from the Figure 3 that the mutations made in the peptide changed its secondary structure. Initially, the peptide, which was a fragment of the ACE2 structure, maintained its alpha-helix structure. The MC/LDS and HO mutations maintained only 29% and 23% sequence identity, respectively. Soon, the 3D structure assumed new conformations. This allowed the peptide to bind to more residues of the spike protein or with a higher binding energy (if we consider the docking score). However, this caused greater mobility of the peptide. Details will be further discussed in the next sections.

### 2.4. The Importance of Protein–Peptide Complexes

In this study, we proposed a method to design peptides that could interact with proteins—in this case, the spike protein from SARS-COV-2. Finding a peptide that interacts with this protein could be a therapeutic option in combating COVID-19. Several articles have reported strategies for inhibiting SARS-COV-2 using peptides. However, this is not a trivial task. Here, we propose a strategy that starts with a peptide produced based on a fragment of the ACE2 protein. Using a strategy based on a genetic algorithm, our strategy (POTTER) tries to find an optimized peptide for this binding.

**Figure 4:**
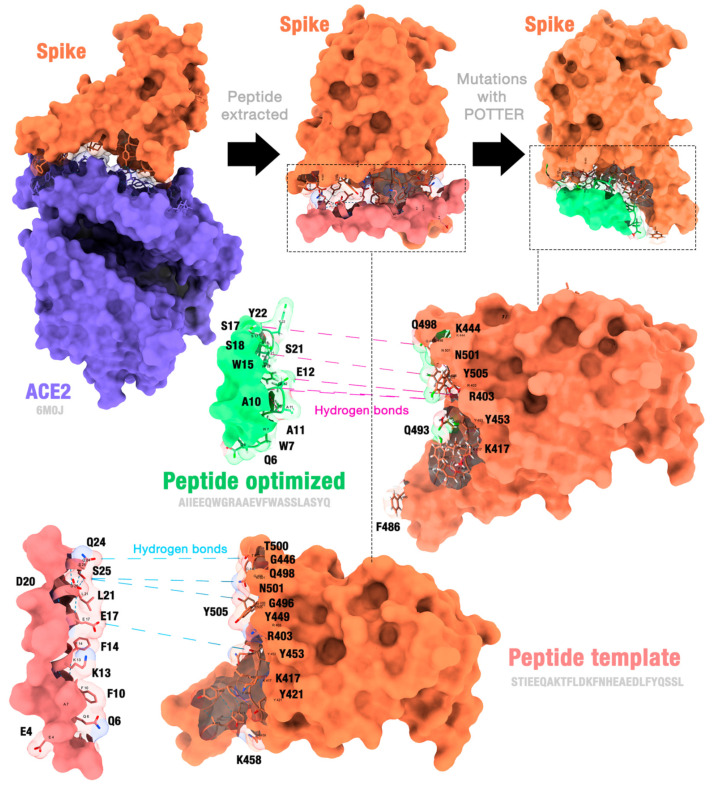
The hydrogen bonds are formed between the peptide template and the receptor, as well as between the manually curated peptide and the receptor. Figure generated using ChimeraX version 1.4.

Figure 4 illustrates our starting point (6M0J structure); how we extracted the template peptide (salmon-colored structure); and, finally, how we obtained an optimized mutant peptide. In this example, the lines indicate hydrogen bonds that connect the interfaces of peptides and proteins.

The selection of the best peptide (i.e., the one most optimized for binding to the spike) was based on several strategies. When performing manual curation (MC), the adopted criterion was the exact location of the peptide at the SARS-CoV-2 RBD–ACE2 interfaces and with the greatest number of ionic interactions, hydrogen bonds, and aromatic stackings, thus ensuring that when binding to the receptor, the peptide model would perform efficient interactions, enabling the inhibition of the spike protein. We considered this parameter more efficient, meeting our expectations. As for the other parameters, the peptide models are also at the interface, but not all of them have the same coverage, as in the case of the lowest docking score (LDS).

The docking score is a criterion that has been widely criticized in the literature [23]. The best pose is not always the one with the lowest docking score, as this score is defined based on criteria established by the docking tool (in this case, PyRosetta). However, despite its limitations, the docking score should not be disregarded. When combined with other strategies, such as analyzing the number of contacts made or manually examining the location of the interaction, the docking score can be a valuable tool in decision-making. In our study, we combined the docking score with contact evaluation and manual curation to select the best peptide based on a comprehensive analysis.

A generally accepted premise is that interacting proteins have a high degree of surface complementarity, but electrostatic complementarity is also observed [24]. Protein–peptide interfaces are complex environments; it would be unfeasible to detail the interactions between residues based on physicochemical analysis. One of the alternatives is to consider the already known studies on structure and protein–peptide interactions, as well as the residue–residue contact preference, that is, deriving interaction potentials from the propensities of residues to interact with each other, as observed in the collection of known structures of protein complexes [25].

It is observed that residues with opposite charges tend to come into contact with each other. The relative orientation of the charged groups of both residues suggests electrostatic attraction between them. This is due to the charged groups that are spatially close to each other in most observed pairs, suggesting that they interact with each other. The attractive interactions between Arg-Glu, Asp-Arg, and Lys-GLu observed at the protein–peptide interfaces of the selected peptides demonstrate the existence of various electrostatic interactions, including salt bridges and hydrogen bonds.

The presence of residues such as tyrosine and tryptophan is observed; these two amino acids have amphipathic side chains with sufficient steric volume and flexibility, allowing the binding interface to form to optimize complementarity, since the hydrophobic effect plays an important role in protein folding and stability.

### 2.5. Comparison with Other Peptide Design Proposals Described in the Literature

Diverse computational and experimental methodologies have been developed to design new peptides or improve existing ones that can inhibit the SARS-CoV-2 spike protein by binding to its receptor-binding domain (RBD). For example, Valiente et al. (2021) employed in vitro methods to create D-peptide inhibitors that mimic the ACE2 α1-binding helix. Their best designs exhibited binding affinities to the RBD of 29 and 31 nM, respectively, and blocked the infection of Vero cells by SARS-CoV-2 with IC50 values of 5.76 and 6.56 μM [26]. In silico methods have also been widely used, with varying degrees of success. Sitthiyotha and Chunsrivirot (2021) utilized molecular dynamics simulations to guide the design of peptides with enhanced binding affinities to the SARS-CoV-2 RBD compared to the human ACE2 receptor, identifying mutations that improve peptide binding to the RBD [27]. Similarly, Sun et al. (2023) employed multiple replica molecular dynamics simulations to investigate the competitive binding of engineered ACE2 proteins, specifically variants 3N39 and 3N94, to the RBD of SARS-CoV-2. By modifying residue types near the binding interface, the engineered ACE2 proteins alter the electrostatic potential distribution and reconfigure the hydrogen bonding network, resulting in modified binding to the RBD [28]. Additionally, Liang et al. [29] evaluated the binding mechanism of inhibitors to the SARS-CoV-2 main protease using MD simulations and suggested that the hot-spot residues include H41, M49, F140, N142, G143, C145, H163, H164, M165, E166, and Q189.

A different computational approach was applied by Robson (2020) using Q-UEL (Quantum Universal Exchange Language) to perform bioinformatics analysis of the SARS-CoV-2 genome. This analysis identified conserved protein sequences for the design of synthetic vaccines and peptidomimetic therapeutics [30]. The study highlighted the KRSFIEDLLFNKV sequence as particularly well-conserved, corresponding to the region around one of the known cleavage sites of the SARS virus that is believed to be necessary for activating the virus for entry into cells [22,30,31]. The proposal includes a specific synthetic vaccine epitope and a peptidomimetic agent. However, the used region is exposed on the surface proteins and is more susceptible to mutations [25,32], which may impact the efficacy of the proposed therapeutics, as coronaviruses recognize different receptors and have entry mechanisms according to the genus they belong to.

In our studies, we present a tool that proposes a design of peptidomimetics based on the SARS-CoV-2–RBD/ACE2 interaction region, a region less susceptible to mutations, ensuring that future therapeutics remain effective over time. Our tool is designed to target specific interactions, using the ACE2 receptor as a starting point. This approach is similar to those proposed in other studies where computational scans are used to create peptides from the analysis of the RBD/ACE2 interaction region, thus minimizing potential interference or unwanted interactions.

### 2.6. Importance of POTTER Parameters

As discussed previously, we performed three case studies with POTTER using different parameters. Determining the best parameters is a complex task. Therefore, we performed case studies varying the number of generations, mutations per generation, and replicates for each docking experiment. Considerably increasing all these parameters has a high computational cost, which can make running POTTER unfeasible.

**Figure 5:**
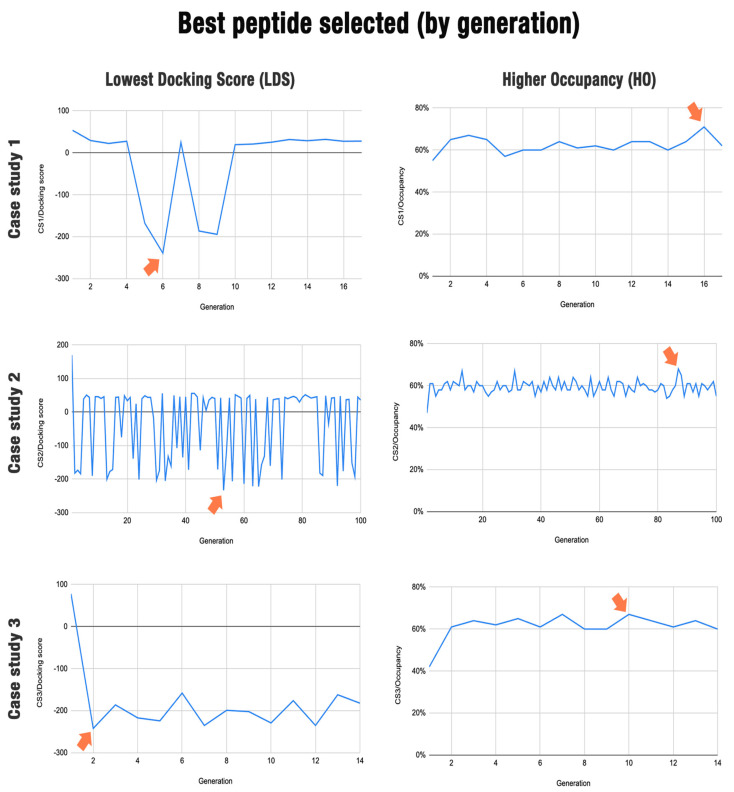
Best peptide selected by generation for case studies 1, 2, and 3. The generation in which each best peptide is obtained is highlighted by a red arrow.

Figure 5 illustrates the docking and occupancy score results for each generation in the three case studies. Note that for docking scores, lower values indicate better interaction. On the other hand, the greater the occupancy (number of residues performing contact), the better the interaction. The red arrow indicates in which generation the best peptide was selected. Note that the peptides with the best results are not found in the latest generation. In each generation, the peptide chosen as the best result is selected for the next generation, but it is not used for comparisons in that generation. Only mutants of this peptide are used. This is due to a behavior established by POTTER to remove the bias of the genetic algorithm in which the algorithm reaches the energy minimum right at the beginning. If the determined discarded peptide is the best peptide in the entire process, the user can select it after the algorithm run is complete.

The figure shows the results achieved in each case study and hits at the effects we can expect from each algorithm parameter. As we can see in the LDS and HO graphs in case study 1 (G = 17, M = 50, R = 50), balancing exploration (number of mutant peptides) with exploitation (number of replicates tested) resulted in inconsistent behavior of the algorithm, with the lowest docking score being achieved relatively early and a slight overall increase in the occupancy over the generations. However, after the tenth generation, the docking score increased, reaching a plateau. The highest occupancy value was only found in the sixteenth generation. Note that we recommend having a low docking score value and a high occupancy value to choose the best peptide.

Case study 2 (G = 100, M = 100, R = 3) shows how using too few replications with few models may lead to unstable results (considering the docking score). While the first case study used 2500 dockings per generation (50 replicates for 50 mutants), this scenario used only 300 (3 replicates for 100 mutations), leading to more exploration but less exploitation, which could lead to good results being missed in some generations. On the other hand, by lowering the computational demand per generation, it is possible to iterate for more generations, which translates into even more exploration. Note that the number of generations may depend on the computational cost. Briefly, fewer replicas considerably reduce the cost of computational processing, which allows the algorithm to reach more generations in less time. Each case study was run for approximately one month using a computer with 32 cores and 96 GB of RAM.

Finally, in case study 3 (G = 14, M = 1000, R = 3), we can see how an exploration-heavy approach can be beneficial in finding the best solutions early. Using 1000 mutants in each generation, we identified good candidates showing minimal docking scores and reasonably good occupation within two generations. It is also worth noting that since results were found early, the identity of the original sequence was also the highest. Supplementary Table S1 shows the other peptides that were not selected for the three case studies.

### 2.7. Insights Obtained by the MD Simulations

**Figure 6:**
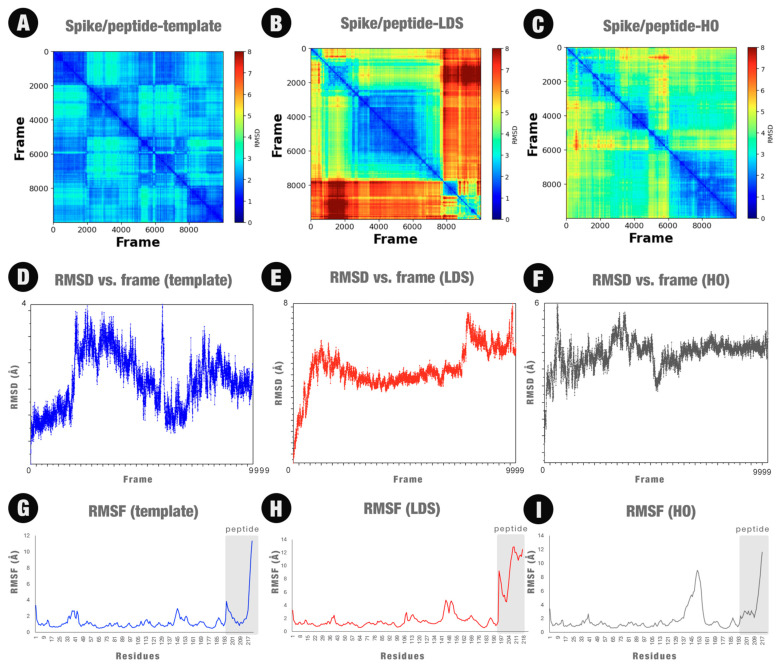
2D-RMSD, RMDS, and RMSF line plots for the three MD simulations: spike complexed with the peptide template (**A**,**D**,**G**, respectively); spike complexed with the LDS peptide (**B**,**E**,**H**, respectively); and spike complexed with the HO peptide (**C**,**F**,**I**, respectively).

The MD simulations demonstrated that the peptides suggested by POTTER for case study 1 were kept in the region closer to the binding site of the spike protein during the 200 ns of simulation. These results are in partial agreement with the other results, indicating that the proposed peptides may be good targets to inhibit the spike protein. However, more in silico, in vitro, and in vivo experiments must be carried out to confirm this. Furthermore, the results of MD simulations must be evaluated carefully. In particular, a comparison of the mobility of the peptide when binding to the protein should be conducted. Figure 6 presents the three case studies’ 2D-RMSD, RMSD, and RMSF line plots.

When we observe the 2D-RMSD graph, we can see the bond between the initial peptide and spike has a lower mobility (Figure 6A) compared to the spike–LDS complex and the spike–HO complex. Although a plateau is not clearly observed in the RMSD vs. frame plot (Figure 6D), the peak in the RMSD variation was 4 Å, which is lower than the peaks obtained from two other systems. Also, we can observe clusters that represent the most adopted conformations based on the bluest squares formed in the region close to the central diagonal. In Figure 6A–C, warm colors indicate greater mobility, while cold colors indicate less mobility.

Lastly, the peptide chosen in manual curation (which has a similar sequence to the LDS peptide) showed a greater variation in RMSD when comparing the 10,000 frames. We can visually confirm the variations in the poses of this peptide when we analyze Figure 3 (middle line). Many changes in protein binding poses can indicate that the peptide may be released from the protein, which is not desired. More molecular dynamics experiments should be carried out in the future to verify this.

## 3. Materials and Methods

### 3.1. Data Collection

The SARS-CoV-2 spike protein structure was retrieved from the Protein Data Bank (PDB) [33]. For this work, we chose the 6M0J structure [2], which contains the SARS-CoV-2 spike protein structure bound to its receptor, the ACE2 protein. By analyzing the structure, we were able to isolate the region of the ACE2 protein that interacts with the SARS-CoV-2 spike protein. We then used PyMOL [34] software to separate this sequence of the ACE2 as a peptide to be used as a template and starting point in the optimization process. We also separated the structure of the spike protein to be used as the target in our algorithm.

With the structures defined, we examined the related literature [2] to identify the receptor-binding domain (RBD) of the SARS-CoV-2 spike protein, which we used to define the binding site. The residues in this region were also used as the reference in counting and classifying contacts with each generated peptide. This approach provided us an additional measure of how well each proposed peptide was binding and effectively blocking the RBD. Structures were analyzed using PyMOL version 2.5 and ChimeraX version 1.4 [35,36].

### 3.2. Algorithm Implementation

**Figure 7:**
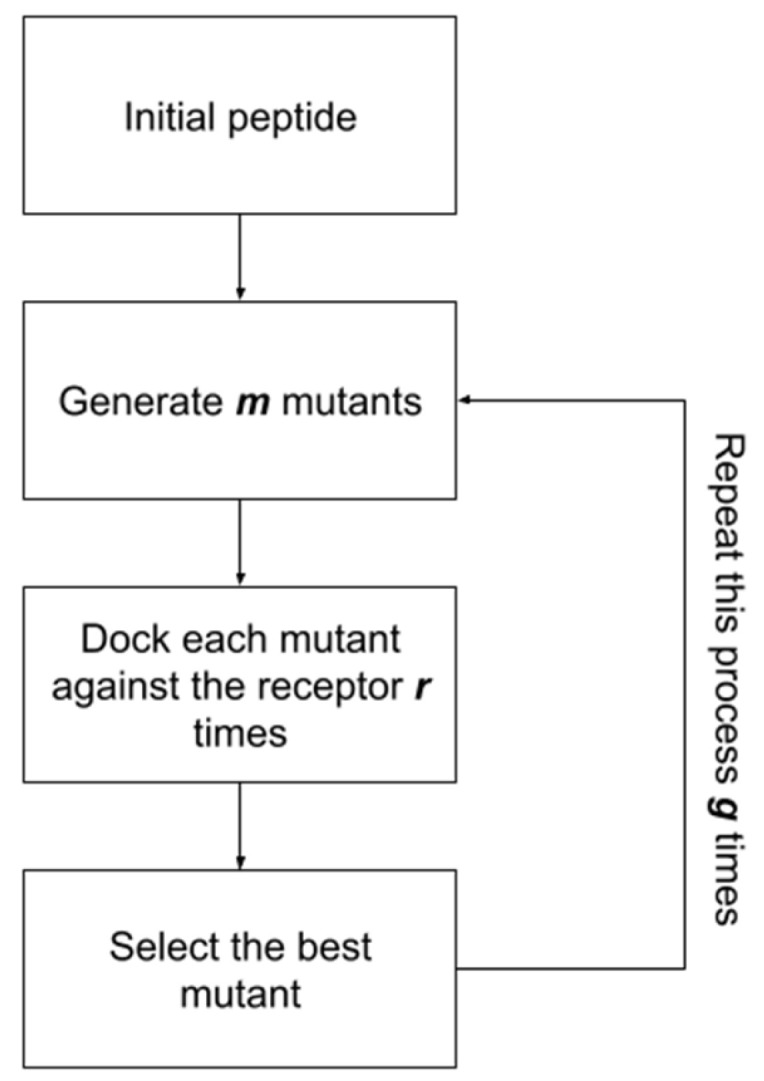
POTTER method workflow.

The algorithm was implemented using Python scripts. Data pre-processing was performed using built-in Python libraries. For molecular docking, we used third-party libraries (details will be presented in the next section). The source code is available at https://github.com/LBS-UFMG/potter (accessed on 23 March 2024). The algorithm methodology is summarized in Figure 7.

### 3.3. Molecular Modeling and Docking

Initially, the peptides were modeled using ColabFold version 1.5.5 [37] with default parameters. For the following executions of the algorithm, we used PyRosetta to perform peptide modeling.

In our study, we employed a range of metrics to assess the potential effectiveness of each peptide in inhibiting the SARS-CoV-2 RBD. A key metric used was the docking score. We adopted the FlexPepDock ab initio protocol [38] for this purpose. This protocol was applied to the structures utilizing PyRosetta [20], a Python package specifically designed for such molecular modeling tasks. We opted to use these tools because of their ability to consider the flexibility of peptides, which is closer to reality than rigid docking.

We started by submitting our template to the redocking process to establish a baseline docking score against which all the subsequent peptides’ scores would be compared. This first experiment was performed once, and 50 models were generated and evaluated. To calculate the score, we used the REF15 scoring function in PyRosetta [38].

Docking was also applied to each new peptide that was created. In each of the applied dockings, N models were generated and scored. The results were stored in a CSV file that would be later enriched with contact information for final classification. All the generated structures (models) were saved for contact analysis.

### 3.4. Contacts

For contact analysis, we used the Signa library (not published yet). Signa considers the definitions pointed out by the nApoli web tool v1.0 [39,40]. For hydrogen bonds, we considered acceptor–donor atom pairs at a distance of up to 3.9 Å. For hydrophobic interactions, we considered apolar atom pairs at a distance of up to 4 Å. For aromatic-stacking interaction, we considered atoms in aromatic chains at a distance ranging from 2 to 4 Å. For attractive interactions, we considered a pair of atoms in residues with different charges, i.e., positive and negative. For repulsive interactions, we considered atoms from amino acids with the same charge, whether positive–positive or negative–negative. For salt bridge interactions, we considered residue pairs that perform both hydrogen bonds and attractive interactions at the same time.

For manual curation, we used the Arpeggio web tool as a complementary method to detect protein–peptide contacts. Additionally, structural alignments were performed using the VTR web tool v1.0 [41,42].

### 3.5. Case Studies

To evaluate the POTTER algorithm, we performed three case studies (Table 3). In all case studies, we started with the peptide sequence STIEEQAKTFLDKFNHEAEDLFYQSSL (also called peptide template). This sequence was obtained from the region in the ACE2 structure that interacts with the spike protein (PDB ID: 6M0J [2]). The sequence was extracted using the PyMOL open-source tool.

### 3.6. Molecular Dynamics

To evaluate the links between peptides and receptors, we performed molecular dynamics (MD) simulations using the NAMD program version 2.14 [43] for the results of case study 1. We prepared the following three systems: (i) a spike protein complexed with the peptide template, (ii) a spike protein complexed with the LDS peptide, and (iii) a spike protein complexed with the HO peptide.

The three systems were prepared using the VMD v1.9 tool [44]. We estimated the protonation states using the APBS-PDB2PQR software (3.6.1) suite [45]. The systems were solvated using a cubic box of 10 Å. Additionally, we added Na^+^ and Cl^−^ ions using the VMD extension tool. After preparation, we performed 10,000 steps of MD simulation for each system using the NAMD tool at a 310 K temperature [46]. Each step consisted of a 0.2 ps frame, totaling 200 ns for each system. Periodic boundary conditions (PBC) were established at a 10 Å cutoff for each border.

RMSD and RMSF analyses were carried out using the VMD tool through the measure rmsd command (“VMD measure documentation”) [44]. 2D-RMDS plots (all-against-all frames) were designed using MDAnalysis [47,48], numpy [49], and Matplotlib libraries [50].

## 4. Conclusions

In this study, we proposed a method to design peptides that can interact with determined proteins. Designing peptides is not a trivial task; however, we hypothesized that our method could be used with other problems. We intend to carry out new case studies in future work. Furthermore, as perspectives for the future, we intend to synthesize the proposed peptides, followed by experiments to verify the interactions between peptides and ligands. We also hope that the computational strategies applied here can be applied to the design of peptides used for pharmaceutical applications.

## Figures and Tables

**Table 1:** Substitutions that were allowed by the POTTER algorithm. Source: adapted from [21].

Amino Acid	Substitution Allowed	Amino Acid	Substitution Allowed
A	E, S, T	M	F, W, Y
C	S, T, V	N	G, D, Q
D	E	P	G
E	A, D	Q	N, E
F	M, W, Y	R	H, K
G	N, P	S	A, T
H	K, R	T	A, I, S
I	L, V	V	A, I, L
K	H, R	W	F, M, Y
L	I, V	Y	F, M, W

**Table 2:** Peptides selected by manual curation, lowest docking score, and highest occupation versus initial peptide.

Case Study	CS1	CS2	CS3
**PDB**	6M0J	6M0J	6M0J
**Receptor**	Spike (6M0J:E)	Spike (6M0J:E)	Spike (6M0J:E)
**Initial peptide sequence**	STIEEQAKTFLDKFNHEAEDLFYQSSL	STIEEQAKTFLDKFNHEAEDLFYQSSL	STIEEQAKTFLDKFNHEAEDLFYQSSL
**Sequence length**	27	27	27
**Occupancy**	55%	47%	42%
**Docking score**	54	170	78
**Parameters**			
**Replicates (per docking)**	50	3	3
**Mutants (per generation)**	50	100	1000
**Generations (max)**	17	100	14
**Manual curation (MC)**			
**Best peptide (MC)**	AIIEEQWGRAAEVFWASSLASYQ	ATVEENSRTYIDHFNRATDDYWATVETFD	TEEQAKTFLDFDLFWQSSLN
**Generation (MC)**	6	14	2
**Docking score (MC)**	−194.71	−177.7	−224.52
**Occupancy (MC)**	41%	37%	33%
**Identity**	29%	29%	57%
**Higher occupancy (HO)**			
**Best peptide (HO)**	ESIDAGFPRAAELFYESALSSMN	TTIEAQAHSMIERWPRDTAEWWTAIATMD	TEENAKSFVDFDLFYQATLQ
**Generation (HO)**	16	87	10
**Docking score (HO)**	1432	302	123
**Occupancy (HO)**	71%	68%	67%
**Identity**	23%	17%	43%

**Table 3:** Input for the case studies.

#	PDB	Initial Peptide Sequence	Replicates	Mutants	Generations
1	6M0J	STIEEQAKTFLDKFNHEAEDLFYQSSL	50	50	17
2	6M0J	STIEEQAKTFLDKFNHEAEDLFYQSSL	3	100	100
3	6M0J	STIEEQAKTFLDKFNHEAEDLFYQSSL	3	1000	14

## Data Availability

The datasets and scripts generated for this study can be found on GitHub at https://github.com/LBS-UFMG/potter (accessed on 23 March 2024).

## Supplementary Materials

The following supporting information can be downloaded at: https://www.mdpi.com/article/10.3390/molecules29071577/s1, Supplementary Table S1. Peptides generated for each case study (with docking scores, occupancy, and identity).
